# New-onset QT prolongation is a novel predictor of mortality in critically ill patients

**DOI:** 10.1186/s13054-019-2514-6

**Published:** 2019-06-21

**Authors:** Yonghe Ding, Ryounghoon Jeon, Linyu Ran, Wenzhi Pan, Feilong Wang, Qiang Li

**Affiliations:** 1 The Affiliated Hospital of Qingdao University, The Biomedical Sciences Institute of Qingdao University, Qingdao University, Qingdao, 266003 China; 2 Department of Cardiovascular Medicine, Mayo Clinic, Rochester, MN 55902 USA; 3 Medical College, Tongji University, Shanghai, 200092 China; 4 Department of Cardiology, Shanghai Institute of Cardiovascular Disease, Zhongshan Hospital, Fudan University, Shanghai, 200032 China; 5 Department of Pulmonary and Critical Care Medicine, Shanghai East Hospital, Tongji University, Shanghai, 200120 China; 6 Department of Emergency, Xinhua Hospital Affiliated to Shanghai Jiaotong University School of Medicine, Shanghai, 200092 China

QT prolongation is associated with increased mortality in different types of patients [1, 2]. QT prolongation is common in critically ill patients [3], and the association between heart rate corrected QT (QTc) and outcome in critically ill patients has raised broad interests most recently [4]. However, the prevalence of new-onset QT prolongation and its significance in these patients was not well studied yet.

Here, we prospectively recruited 505 consecutive ICU patients without known previous QT prolongation to evaluate the risk factors for new-onset QT prolongation and the prognostic value of QTc calculated by different methods. The baseline clinical and laboratory characteristics of subjects were shown in Table 1. The mean Bazett QT interval was 413.6 ± 33.8 ms. New-onset QT prolongation occurred in 99 patients (19.6%). This occurrence is about 200-fold higher than that in the general population [5]. Intriguingly, the occurrence of nonthyroidal illness syndrome (NTIS) is significantly higher in patients with QT prolongation than those without (Table 1), indicating that NTIS might be a risk factor of QT prolongation. Indeed, multivariate linear regression showed that QTc was independently associated with NTIS, heart rate, level of serum potassium, gender, and estimated glomerular filtration rate (eGFR).

**Table 1:** Clinical and laboratory characteristics of subjects

	All	Normal QT (*N* = 406)	QT prolongation (*N* = 99)	*p*
Age (years)	63.7 ± 18.2	62.8 ± 18.5	67.4 ± 16.6	0.20
Male (%)	305 (60.4)	237 (58.4)	68 (68.7)	0.06
Heart rate (BPM)	85.2 ± 20.4	83.7 ± 20.8	91.3 ± 17.5	0.01
Positive cTNT (%)	52.5	46.5	76.8	< 0.001
LogNT-proBNP	2.3 ± 0.7	2.2 ± 0.7	2.6 ± 0.6	0.91
Na+ (mmol/L)	140.2 ± 5.7	140.0 ± 5.5	141.0 ± 6.5	0.15
K+ (mmol/L)	3.9 ± 0.6	3.9 ± 0.6	3.8 ± 0.7	0.10
Cl- (mmol/L)	104.7 ± 6.4	104.6 ± 6.0	105.5 ± 7.8	0.04
Ca2+ (mmol/L)	2.08 ± 0.21	2.10 ± 0.19	2.02 ± 0.25	0.001
FBG (mmol/L)	7.53 ± 3.28	7.36 ± 3.02	8.26 ± 4.15	0.02
eGFR (mL/min/1.73 m^2^)	86.7 ± 44.1	91.6 ± 42.9	66.3 ± 43.5	0.49
CKD grade^#^	1.95 ± 1.13	1.80 ± 1.01	2.56 ± 1.35	< 0.001
APACHE- II (points)	15.0 ± 8.4	14.2 ± 8.0	18.7 ± 9.2	0.006
TT3 (nmol/L)	0.92 ± 0.45	0.96 ± 0.48	0.73 ± 0.25	0.004
TT4 (nmol/L)	86.4 ± 30.1	88.7 ± 30.9	77.4 ± 24.8	0.19
FT3 (pmol/L)	3.44 ± 1.10	3.52 ± 1.18	3.15 ± 0.59	0.17
FT4 (pmol/L)	15.5 ± 4.8	15.5 ± 5.1	15.4 ± 3.6	0.67
TSH (IU/mL)	1.34 ± 1.35	1.34 ± 1.28	1.32 ± 1.61	0.06
^#^NTIS (%)	59.3	55.0	76.3	< 0.001

*BPM* beats per minute, *FBG* fasting blood glucose, *eGFR* estimated glomerular filtration rate, *CKD* chronic kidney disease, *APACHE II score* Acute Physiology and Chronic Health Evaluation II score. *TT3* total triiodothyronine, *TT4* total thyroxine, *FT3* free triiodothyronine, *FT4* free thyroxine, *TSH* thyroid-stimulating-hormone, *rT3* reverse triiodothyronine, *NTIS* nonthyroidal illness syndrome

^#^NTIS: Euthyroid patients with fT3 decreased below the normal range (< 3.5 pmol/L) during critical illness

There was a significantly graded increase in mortality rate across increasing QTc quintile (*p* = 0.004) (Fig. 1a). The overall mortality rate in patients with a new-onset QTc prolongation is more than two times higher than those patients without (22.2% vs 9.6, OR = 2.69, *p* = 0.001) (Fig. 1b). Multivariate logistic regression showed that QT prolongation is still independently associated with ICU mortality even after adjusted for age and gender (*p* = 0.001, 95% C.I., 1.51–4.79). However, QT prolongation is no longer a predictor of ICU mortality if APACHE-II score was further adjusted (*p* = 0.329), likely due to that QTc itself is strongly associated with APACHE-II score (*r* = − 0.235, *p* < 0.001).

**Fig. 1:**
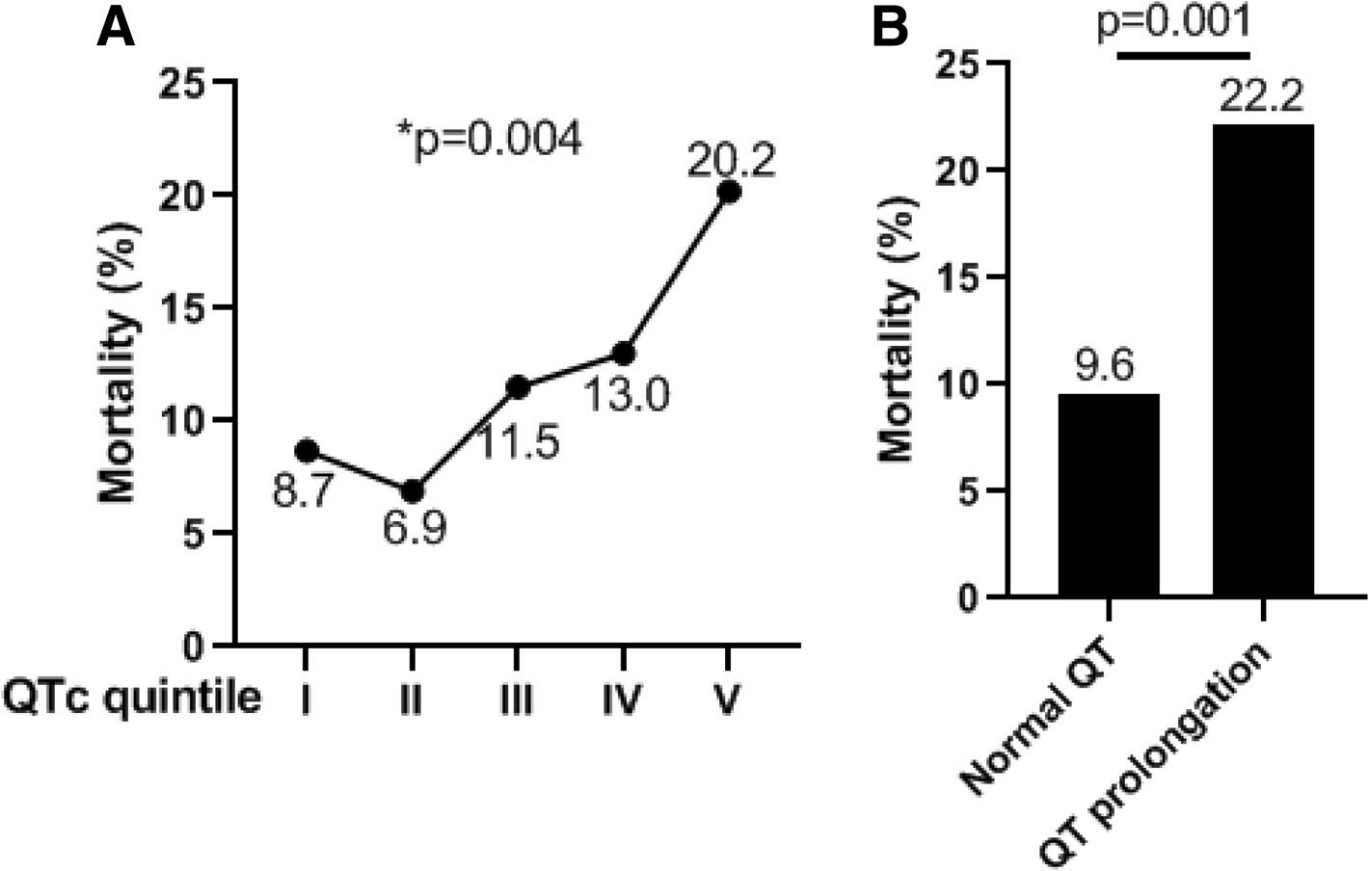
**a** ICU mortality rates among patients with different QTc quintile. *Linear-by-Linear Association by chi-square test. **b** Overall ICU mortality rates among patients with or without QT prolongation

As Bazett’s formula can over-correct QT at high heart rates and under-correct it at low heart rates, we then evaluated the prognostic value of QTc calculated using additional formulas including Fridericia’s, Framingham’s, and Hodges’s. We found that patients in quintile 5 have significantly higher mortality than patients in the combination of quintiles 1–4 regardless of which formula was used (all *p* < 0.05).

In summary, QT prolongation determined by baseline ECG can serve as a novel indicator of the severity of illness in critically ill patients. NTIS is a new risk factor of QT prolongation in critically ill patients.

## Data Availability

The datasets used and/or analyzed during the current study are available from the corresponding author on reasonable request.

## Abbreviations

APACHE-II score: Acute Physiology and Chronic Health Evaluation II score
eGFR: Estimated glomerular filtration rate
ICU: Intensive care unit
NTIS: Nonthyroidal illness syndrome
QTc: Heart rate-corrected QT
